# Wound Dressing Modifications for Accelerated Healing of Infected Wounds

**DOI:** 10.3390/ijms24087193

**Published:** 2023-04-13

**Authors:** Vladyslav Vivcharenko, Marta Trzaskowska, Agata Przekora

**Affiliations:** Independent Unit of Tissue Engineering and Regenerative Medicine, Medical University of Lublin, Chodzki 1, 20-093 Lublin, Poland; vladyslav.vivcharenko@umlub.pl (V.V.); marta.trzaskowska@o2.pl (M.T.)

**Keywords:** antibacterial dressing materials, biomaterials, essential oils, nanoparticles, polyphenols, curcumin, skin regeneration

## Abstract

Infections that occur during wound healing involve the most frequent complications in the field of wound care which not only inhibit the whole process but also lead to non-healing wound formation. The diversity of the skin microbiota and the wound microenvironment can favor the occurrence of skin infections, contributing to an increased level of morbidity and even mortality. As a consequence, immediate effective treatment is required to prevent such pathological conditions. Antimicrobial agents loaded into wound dressings have turned out to be a great option to reduce wound colonization and improve the healing process. In this review paper, the influence of bacterial infections on the wound-healing phases and promising modifications of dressing materials for accelerated healing of infected wounds are discussed. The review paper mainly focuses on the novel findings on the use of antibiotics, nanoparticles, cationic organic agents, and plant-derived natural compounds (essential oils and their components, polyphenols, and curcumin) to develop antimicrobial wound dressings. The review article was prepared on the basis of scientific contributions retrieved from the PubMed database (supported with Google Scholar searching) over the last 5 years.

## 1. Introduction

Any disorder of skin function or its healthy structure due to injury, thermal trauma, genetic disorders, or even surgical interventions leads to wound formation [1]. Among all causes of worldwide disability, skin diseases are located in the fourth place, resulting in high levels of harm to patients’ bodies and minds and simultaneously generating huge economic burdens for society [2]. The skin accounts for about one-sixth of the whole body’s weight and covers around 3000 square inches of its surface. Therefore, it is the organ of the human body most exposed to various external threats [3]. Skin plays a key role in sensing the environment, maintaining thermal and physicochemical homeostasis, providing active and passive defense, acting as a container of important nutrients, and responding to injury and trauma. Maintaining the main skin functions is required not only for preventing trauma, but also for effective wound repair [4]. The physiological regulation process of skin wound healing requires the intricate synchronization of different mediators and cell types [5,6]. The interactions between cytokines, chemokines, extracellular matrices, cells, growth factors, and other regulatory molecules are crucial in wound closing during the healing process [7,8]. On the other hand, infection is the key factor and the most frequent complication in the field of wound care which not only inhibits the whole process, but also leads to non-healing wound formation [9]. 

## 2. The Effect of Bacterial Infection on the Wound-Healing Process

Both acute and chronic skin injuries are healed in a process that consists of four successive phases: hemostasis, inflammation, proliferation, and remodeling [10]. The effect of bacterial contamination on wound healing depends on various factors and causes different disturbances depending on the stage of wound repair [11]. The final goal of the normal wound healing process is to eliminate the invading microorganisms and clean the wound of damaged cells, which allows the restoration of the skin’s most important function—acting as a barrier that protects the body against the external environment. Disrupting, extending, or stopping the process at any of the repair steps results in impaired healing and chronic wound formation [12].

### 2.1. Hemostasis Phase

After skin injury, coordinated phases are activated by various inter- and intracellular biochemical pathways in order to re-establish tissue integrity and homeostasis. Different cells, such as fibroblasts, keratinocytes, neutrophils, endothelial cells, macrophages, monocytes, dendritic cells, and lymphocytes, together with inflammatory pathways and the coagulation cascade, are also involved [7]. After the injury, smooth muscles, which are located in the round layer of the vessel wall, contract, causing the spasm of the damaged arterial vessel. Reduced blood flow caused by the constriction of the arterioles leads to tissue hypoxia, which in turn causes the production of nitric oxide and vasoactive metabolites that begin the relaxation and vasodilation of the arterial vessels [13]. Intrinsic and extrinsic pathways of the clotting cascade and platelet activation are the subsequent three key mechanisms that are responsible for the prevention of further blood loss [14]. As a result, the hemostatic effect is obtained due to platelet aggregation and fibrin clot formation, transferring the wound into an inflammatory stage [15]. The presence of bacteria in an open wound exerts a negative impact on hemostasis, which may be related to endotoxin and exotoxin presence, tissue-destroying (lytic) enzymes, or antiphagocytic effects [16].

### 2.2. Inflammation Phase

An increased number of inflammatory cells in the extracellular space around the wound results in the wound’s characteristic red and warm appearance. The prevention of bacterial contamination and infection is the main goal of this phase of wound healing. The stage starts with wound infiltration by neutrophils. The process takes place within an hour, and then for two days the neutrophil concentration remains constant [13]. Non-viable tissue and bacteria are then digested by caustic proteolytic enzymes, antimicrobial proteins, and reactive oxygen species (ROS) released by neutrophils [17,18]. Nevertheless, some of these microbicidal molecules synthesized by neutrophils have cytotoxic effects on host tissues and can lead to tissue necrosis, impairing healing [19]. Macrophages/monocytes and leukocytes, which are essential for wound healing, are the next cells present in the wound bed. Macrophages are responsible for the synthesis of numerous cytokines and enzymes, including (I) tumor necrosis factor alpha (TNF-α) and interleukins (ILs) for fibroblast stimulation and angiogenesis promotion, (II) collagenases for wound debriding, and (III) tissue growth factor (TGF) for keratinocyte stimulation [18]. Incomplete wound decontamination may result in a prolonged inflammation phase due to the maintenance of a high level of pro-inflammatory cytokines (IL-1 and TNF-α). Infections caused mainly by Gram-negative bacteria are responsible for a significant depletion of different factors of the complement cascade, reducing its effectiveness [20]. The long-term condition described above also increases matrix metalloproteinase (MMP) production, which in turn leads to protease imbalance. Consequently, incomplete elimination of infection may easily lead to chronic wound formation [21,22]. At the end of the inflammatory phase, collagen fibers start to appear at the margins of the wound [23].

### 2.3. Proliferation Phase

The formation of granulation tissue composed of fibroblasts, macrophages, and a new capillary network is a principal step in the proliferative phase. Besides angiogenesis and collagen deposition, the proliferation phase is also characterized by epithelialization stimulated by pro-inflammatory cytokines (TNF-α and IL-1) [24]. Proper wound healing is dependent on the supply of appropriate amounts of nutrients that enhance tissue deposition. Due to this fact, capillary migration and further angiogenesis are critical for appropriate wound healing [18]. Macrophages and activated platelets produce TGF-α and epidermal growth factor (EGF), stimulating the proliferation of epithelial cells. Keratinocytes that are stimulated by IL-6 and keratinocyte growth factors (KGF-1 and KGF-2) synthesized by fibroblasts migrate to the wound area and differentiate in the epidermis [23]. Additionally, macrophages, vascular smooth muscle cells, and polymorphonuclear leukocytes synthesize a vascular endothelial growth factor (VEGF) that promotes endothelial cell proliferation and stimulates angiogenesis [25]. The predominant proliferating cells during this phase are fibroblasts and endothelial cells. Endothelial cells are mainly responsible for the formation of new capillary tubes. In turn, the fibroblasts surrounding the wound become activated by platelet-derived growth factor (PDGF) and EGF derived from platelets and macrophages and begin to proliferate and synthesize type III collagen [26]. Activated by released proteases, TGF-β induces fibroblasts to decrease the production of MMPs, enhance the synthesis of type I collagen, and increase production of cell adhesion proteins [8,27]. Finally, myofibroblasts (fibroblasts transformed with macrophage-secreted TGF-β1) cause wound contraction and, consequently, complete wound closure [28]. Considerable bacterial colonization causes significant retardation of the proliferative phase. Bacterial wall products and components, such as lipopolysaccharide (LPS) and Braun lipoprotein (BLP), significantly reduce endothelial growth factor receptor expression. Consequently, this results in the suppression of endothelial cell migration and proliferation in both normoxic and hypoxic conditions [25]. Major bacterial colonization results in the formation of biofilm that is resistant to eradication and removal [29].

### 2.4. Remodeling Phase

Deposition of type I collagen, which is stronger than type III collagen, is the main feature of the remodeling phase. Wound strength depends on the amount of deposited matrix in the wound bed. Nevertheless, excessive type I collagen synthesis may result in keloid or hypertrophic scar formation [30]. Due to the constant remodeling of the matrix, the collagen synthesis and its breakdown is a continuous process. Its end occurs when the equilibrium state of these two processes is achieved [23]. Various conditions in the wound environment, such as changes in pH due to wound healing, influence the activity of proteinases which are responsible for coordinated wound healing [31]. MMPs are mainly responsible for wound remodeling, namely, balance between collagen synthesis and its degradation. The appropriate equilibrium between MMPs’ activity and their inhibitors is crucial for the normal wound repair process [32]. Type III collagen is degraded by different MMPs released by macrophages and fibroblasts and replaced with type I collagen. In the next stage, type I collagen is reorganized into paralleled fibrils, which in turn enables low cellularity scar formation [8,33]. During the remodeling phase, the bacterial infection causes limited type I collagen production and its degradation by multiple endotoxins, disturbing the reinforcement of the wound [20,34]. A short summary of the effects of bacterial infection on the wound healing phases is presented in Table 1.

### 2.5. Stages of Wound Infection

**Figure 1:**
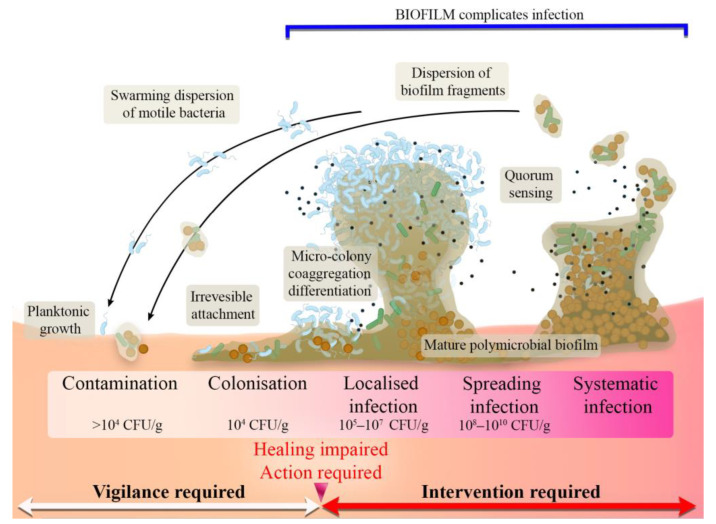
Scheme presenting the wound infection continuum (reproduced from [38] with permission from the Wounds Group, OmniaMed Communications; own modifications were introduced based on information found in [38,39,40]).

The diversity of the skin microbiota and the wound microenvironment can favor the occurrence of skin infections, thereby slowing down or hindering the skin repair process. When the continuity of the skin is disrupted, the native skin flora gain access to a warm and nutrient-rich environment, changing into more aggressive microbial types [35]. Microorganisms, such as *Streptococcus pyogenes*, *Pseudomonas aeruginosa*, *Escherichia coli*, and *Staphylococcus aureus*, are the main strains responsible for bacterial wound invasion. Once the damaged skin tissue ceases to function as a protective barrier, the wound may be simply colonized, leading to potential infection, usually resulting in chronic wound formation [36]. The pathogenic impact on wounds is strictly dependent on advanced and dynamic interactions between pathogens, the host immune system, and the environment surrounding the wound. As a result, a wound infection continuum can be divided into different stages as follows (Figure 1):

**Contamination** that is characterized by a low level of non-proliferating microorganisms. Natural flora, which are typical in this stage of wound contamination, fail to induce the immune response or do not delay healing [37].

**Colonization** that is also characterized by no immune response and normal healing without any delay. The bacteria possess a limited proliferation rate; nevertheless, antimicrobials are not necessary at this stage [37].

**Local infections** that appear when bacteria start to proliferate faster, penetrate deeper into the wound, and begin to initiate host response. This stage is characterized by subtle symptoms and requires medical intervention to stop the development of more serious infection [37].

**Spreading infection** that begins at the moment when bacteria numbers increase and pathogens start to invade the tissue surrounding the wound. This increases the virulence of the infection, simultaneously delaying wound healing and causing wound breakdown and erythema [37].

**Systemic infection** that is the last and the most dangerous stage of the wound infection continuum. Bacteria spread through the lymphatic or vascular routes, usually leading to sepsis and organ dysfunction [37].

From the point of view of preventing wound infection, the most important step is to disable the transition from the colonization phase to local wound infection. According to different studies, it is suggested that wound contamination loaded with more than 10^4^ CFU/g impairs healing, increasing the risk of local infection [40]. Lack of intervention may result in the progression of infection and biofilm formation, increasing resistance to immunological, antimicrobial, and chemical factors [29].

## 3. Antimicrobial Biomaterials

Since the warm, moist, and highly nutritious environment presented in the wound bed provides perfect conditions for microorganisms’ growth, antimicrobial bioactive and interactive dressings play a key role in the modern treatment of open wounds [41]. The golden age of antibiotics research resulted in the discovery of many new chemical compounds that were relatively effective in bacterial infection treatment [42]. Nevertheless, due to the overuse of antibiotics and thus the growing number of drug-resistant strains, their effectiveness has been increasingly lowered [43]. It is justified to search for new compounds that could be used to combat bacterial infections, but these kind of studies are very costly, time-consuming, and often do not show sufficient effectiveness [42]. According to the available literature, antimicrobial dressing materials can be classified into four main types: (I) antibiotic-based materials; (II) materials loaded with nanoparticles; (III) materials containing cationic organic agents; and (IV) others, including materials loaded with antimicrobial plant-derived compounds [44]. The present article focuses on all the abovementioned antimicrobial wound dressings. The review article was prepared on the basis of scientific contributions retrieved from the PubMed database (supported with Google Scholar searching) over the last 5 years.

### 3.1. Antibiotic-Loaded Dressing Materials

**Figure 2:**
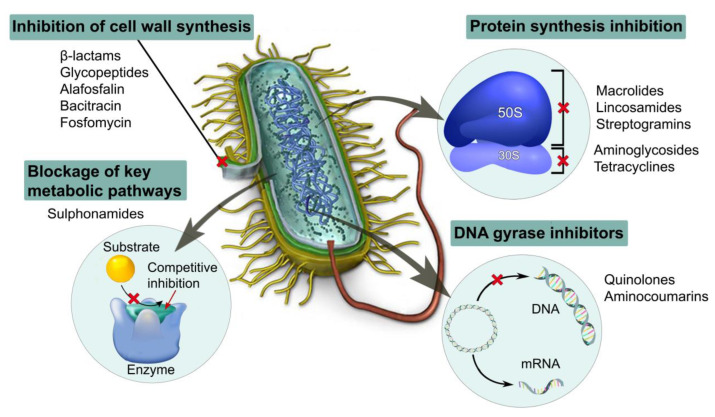
Possible mechanisms of bacterial activity disruption caused by antibiotics (scheme prepared based on the information presented in [46,47]).

The antibiotics loaded into a material may act according to one of the following scenarios: (I) provide inhibition of bacterial cell wall synthesis; (II) provide blockage of key metabolic pathways; (III) provide interference in protein synthesis; or (IV) provide inhibition of nucleic acid synthesis [45]. A schematic representation of antibiotic action mechanisms is presented in Figure 2.

The most common antibiotics used for the production of biomaterials with antimicrobial properties are gentamicin [48,49,50], tetracycline [51,52,53], ciprofloxacin (CIP) [54,55], and sulfadiazine [45,56,57,58,59]. Currently, there are many scientific articles describing wound dressing materials loaded with antibiotics. The latest reports concerning the evaluation of the antimicrobial properties of antibiotic-enriched biomaterials dedicated to wound healing are summarized in Table 2.

In our previous work, the bactericidal action of gentamicin-loaded biomaterial was confirmed. Curdlan/agarose sponge-like biomaterial was loaded with 0.2% wt% gentamicin and evaluated against *S. aureus* and *P. aeruginosa.* In a direct contact test, strong bactericidal activity was noted for both bacterial strains. The same results were observed in a bacterial growth inhibition test, where dressing material with gentamicin significantly reduced *S. aureus* and *P. aeruginosa* growth compared to the control [48]. Bakhsheshi-Rad et al. [60] developed a chitosan-alginate nanofiber dressing containing various concentrations of gentamicin (0 to 10 wt%). All tested concentrations showed antibacterial activity against two strains: *S. aureus* and *E. coli*. A larger inhibition zone was observed with higher antibiotic concentrations. Nevertheless, the most supportive antibiotic concentration that positively influenced wound closure and had a positive impact on biomaterial mechanical and physicochemical properties was noted in the case of 3 wt% gentamicin. The combination of gentamicin and biomaterial based on natural rubber also revealed high antibacterial activity against *S. aureus* and *P. aeruginosa* [61]. Each film was composed of natural rubber and gentamicin and optionally included glycerin, triethyl citrate, xanthan gum, or a mixture of listed compounds. All produced biomaterials that contained gentamicin were characterized by high inhibition zones compared to negative controls. Nevertheless, only the NRTX sample (film based on natural rubber, gentamicin, triethyl citrate, and xanthan gum) revealed a similar inhibition zone to a positive control (1 mg/disc gentamicin), indicating its great antibacterial properties. In another study, the authors used tetracycline hydrochloride to improve the antibacterial properties of fungal chitosan-based biomaterials [62]. An in vitro antibacterial activity test revealed that chitosan sponges containing tetracycline possessed higher inhibitory actions against tested bacteria. Nevertheless, the highest antibacterial properties were noted in the case of biomaterial composed of both tetracycline and *aloe vera.* In turn, CarvalhoAlavarse et al. [63] evaluated nanofiber scaffolds based on polyvinyl alcohol (PVA) and chitosan loaded with tetracycline. Synthesized biomaterials were evaluated against Gram-positive *S. aureus* and *Staphylococcus epidermidis* and Gram-negative *E. coli*. The conducted research proved that tetracycline-loaded nanofibers possessed a higher inhibitory effect on *E. coli*, *S. epidermidis*, and *S. aureus* than PVA/chitosan and PVA material. To provide better antibacterial activity, Elashnikov et al. [54] loaded 10 wt% of ciprofloxacin into Poly(N-isopropyl-acrylamide-co-acrylamide)/Polycaprolactone-based biomaterials. All tested samples were produced using different concentrations of Poly(N-isopropyl-acrylamide-co-acrylamide) (PNIPAm-co-AAm). Antibacterial activity was estimated on the basis of disc diffusion tests and evaluation of bacterial attachment. The obtained results confirmed that all tested dressing materials were characterized by antibacterial activity against *E. coli* and *S. epidermidis*. Nevertheless, higher concentrations of PNIPAm-co-AAm resulted in a significant decrease in bacterial adhesion to the tested nanofibers, which is very important, taking into account the potential application of the materials as wound dressings. In turn, Cacicedo et al. [55] modified chitosan/cellulose material with ciprofloxacin. The authors used a modified disc diffusion method to determine inhibition halos against *P. aeruginosa* and *S. aureus.* Due to some antibacterial properties of chitosan, good inhibitory effects were observed in both films with and without ciprofloxacin. Nevertheless, a synergic increase in antibacterial properties was observed after antibiotic incorporation. Khan et al. [56] modified cellulose acetate nanofibers with different concentrations of silver-sulfadiazine (0.125, 0.25, 0.37, and 0.5 wt%). Small initiation of the antibacterial zone via the agar disc diffusion method was noted in the case of the lowest concentration of silver-sulfadiazine. For *E. coli*, the measurements were two times higher than for *Bacillus subtilis* (1 mm inhibition zone), which might have been related to the difference in the cell wall structures. Nevertheless, 0.25 and 0.5 wt% were the most promising concentrations, with the inhibition zones exceeding 18 mm for both strains. In another study, chitosan film was enriched with silver sulfadiazine-impregnated zeolite [57]. The authors evaluated the antimicrobial activity of the polymer films incorporated with the antibiotic against *E. coli*, *S. aureus*, *P. aeruginosa*, and *Candida albicans.* Chitosan films with the addition of silver sulfadiazine revealed better antimicrobial activity against *C. albicans*. According to the authors, lower activity against bacterial strains (*P. aeruginosa*, *E. coli*, and *S. aureus*) was caused by relatively low antibiotic concentrations. Nevertheless, Gram-negative strains were more susceptible compared with Gram-positive ones. No differences in the case of *S. aureus* viability were noted between the tested films. In turn, Ullah et al. [59] evaluated the antibacterial properties of zein-based nanofiber mats loaded with different concentrations of silver sulfadiazine (0.3, 0.4, 0.5, and 0.6 wt%). Via the disc diffusion method, zone inhibition of the bacteria (*E. coli* and *B. subtilis*) was observed with both strains in comparison to the control. The highest antibacterial activity of the nanofiber mat was noted for the sample with the highest drug concentration. It was also noted that mats with 0.3 and 0.4 wt% of silver sulfadiazine were degraded only by *B. subtilis* due to insufficient amounts of drug, indicating a lack of antibacterial activity for the used concentrations against the tested Gram-positive bacterium. Except for the above-mentioned agents, there are also other groups of antibiotics used to improve the antimicrobial properties of materials dedicated to skin regeneration [45]. Naeimi et al. [64] used vancomycin, which belongs to the class of glycopeptides, in chitosan/PVA/polyethylene glycol (chitosan/PVA/PEG) hydrogel synthesis. The antibacterial properties of the produced hydrogels were tested against *S. aureus* with the disc diffusion method. The inhibition zones for both chitosan/PVA and chitosan/PVA/PEG biomaterials were higher compared to unloaded hydrogel, indicating better antibacterial properties. In order to improve the bactericidal effect, beta-lactam antibiotics were also used, although in a narrower range. Özkahraman et al. [65] used sodium ampicillin in their research. All biomaterials modified with the abovementioned drug were evaluated against ampicillin-sensitive *E. coli* and *S. aureus* in agar disc diffusion tests. Gelatin/ampicillin and gelatin/gellan gum/ampicillin materials showed bactericidal action against both strains, with zones of inhibition of 14–19 mm. In the case of the gellan gum/ampicillin hydrogel, an inhibition zone was noted only for *S. aureus*. Despite the general availability and good effectiveness of various antibiotics in the treatment of wound infections, their use may lead to the emergence of new mechanisms of bacterial drug resistance [66,67]. According to scientific reports, about three-quarters of infectious bacteria are resistant to at least one antibiotic used for infection treatment nowadays [68]. Therefore, it seems reasonable to search for and use other compounds that have antimicrobial activity and do not lead to the extension of the problem of drug-resistant strains.

### 3.2. Nanoparticle-Enriched Dressing Materials

Another important problem in addition to drug resistance is the formation of biofilm by bacteria, which can prevent the penetration of drugs into the infected wound bed [69]. Due to high specific physicochemical, biological, and optical properties, nanoparticles (NPs) have been increasingly used in regenerative medicine [70]. In addition, NPs have emerged as promising alternatives to the antibiotic therapy of multidrug-resistant (MDR) bacterial infection of wounds [71].

#### 3.2.1. Metallic Nanoparticles

Nanoparticles have become a valid tool for the treatment and regeneration stimulation of different types of wounds. Potential therapeutic effects have been observed in the case of gold (Au), silver (Ag), platinum (Pt), copper oxide (CuO), iron oxide (Fe_3_O_4_), and zinc oxide (ZnO) nanoparticle usage [72]. Antimicrobial activities that are inherent to metallic NPs are mainly caused by their high surface areas, particle shapes, and small sizes. Moreover, the high ability of NPs to generate reactive oxygen forms contributes to their high antimicrobial activity [73]. The studies focused on the positive impact of metallic NPs on biomaterials’ antimicrobial properties are summarized in Table 3.

Among metallic nanoparticles, Ag-NPs are known to be some of the most effective, especially in the case of nosocomial strains of multidrug-resistant (MDR) microorganisms [85]. Alipour et al. [74] modified electrospun nanofibers containing PVA, polyvinylpyrrolidone (PVP), pectin (PEC), and mafenide acetate (MF) with different concentrations of Ag-NPs (0.2, 0.5, and 0.7 wt%). The antibacterial activity of the produced nanofibers was evaluated against Gram-positive *S. Aureus* and Gram-negative *E. coli* and *P. aeroginosa* strains using a disc diffusion method. According to the obtained results, the addition of Ag-NPs to the PVA/PVP/PEC/MF nanofibers resulted in the appearance of an inhibition zone which was about 2.5–5 mm. It should be noted that higher activity was noted against Gram-negative bacteria, which was related to intrinsic differences in cell walls. In another study, Ding et al. [75] incorporated Ag-NPs into a bilayer chitosan composite to inhibit microbial invasion. The antibacterial activities of the produced sponges were evaluated using the inhibition zone method. Tested samples containing Ag-NPs exhibited significantly higher antibacterial activities compared to the control. The effectiveness of Ag-NPs was also proven in the work of Li et al. [76]. The authors produced lignin-based polyol foams loaded with Ag-NPs and estimated their antibacterial properties using the plate counter method. According to the obtained results, samples enriched with Ag-NPs significantly reduced the time required to achieve an antibacterial rate above 99% compared to the control. A sample with a high concentration of Ag-NPs was able to reduce *E. coli* and *S. aureus* viability below 99% after 1 and 4 h, respectively. In another study, the authors produced different hydrogels incorporated with Au-NPs (poly ethylene glycol (PEG)-Au nanorods, polyallylamine hydrochloride (PAH)-Au nanorods, and polyacrylic acid (PAA)-Au nanorods) to maximize wound healing efficiency [77]. Au-NPs were selected due to their potent antimicrobial activity and promising candidature for the eradication of wound infections [86]. According to antibacterial activity evaluation, all of the tested hydrogels showed a high log reduction in bacterial viable counts (>99% for both strains) compared to the non-treated control. The effectiveness of Au-NPs was confirmed in in vitro and in vivo studies in the work of Arafa et al. [78]. The in vitro antibacterial evaluation of Pluronic^®^F127/Au gel showed more rapid antibacterial activity compared to Pluronic^®^F127/hydroxypropyl methylcellulose/Au sample. Nevertheless, for both mentioned samples, no bacteria were detected on the fifth day in in vivo studies using rat models with infected burn wounds. It should be noted that in the case of the positive control (silver sulfadiazine), bacterial growth was observed until day 7. Except for Ag-NPs and Au-NPs, there are also articles that describe the effectiveness of CuO-NPs. Balcucho et al. [79] synthesized polycaprolactone/CuO-NP wound dressings using three different concentrations of CuO-NPs (0.05, 0.07, and 0.1 wt%). The antibacterial activity of potential wound dressings was estimated against methicillin-resistant *S. aureus* (MRSA). In the case of the two highest NPs concentrations (0.07 and 0.1 wt%), no viable MRSA cells were noted after 24 h of bacterial exposure to the biomaterial. Nevertheless, the biomaterial with 0.05 wt% CuO-NPs was characterized by the same MRSA growth as the control (pure polycaprolactone material). Karuppannan et al. [80] also proved the effectiveness of using CuO-NPs. The authors infused polycaprolactone/gelatin (PCL/Gel) electrospun nanofibers with CuO-NPs to improve material utility as an antimicrobial wound dressing. In Kirby–Bauer disc diffusion assays, no bactericidal activity against *E. coli*, *P. aeruginosa*, multidrug-resistant *S. aureus* (MDRSA), or *S. aureus* was noted for CuO-NP-free material. Instead, PCL/Gel/CuO-NPs biomaterial caused a statistically significant reduction in the viability of all tested bacterial strains. In the last decade, Fe_3_O_4_-NPs have also been used to improve biomaterials’ antimicrobial properties [87,88]. Paydayesh et al. [81] incorporated various amounts of Fe_3_O_4_-NPs (5, 10, and 15 wt%) into nanocomposite hydrogels based on poly(hydroxyl ethyl methacrylate) (pHEMA) using radical polymerization. A microbe penetration test was conducted for the sample with the highest Fe_3_O_4_-NP concentration. It was revealed that hydrogel with 15 wt% of Fe_3_O_4_-NPs was impenetrable for bacteria. Inhibition zones of 8 mm and 11 mm around the sample were noted for *E. coli* and *S. aureus*, respectively. Similarly, Chircov et al. [82] evaluated the antimicrobial activity against *S. aureus*, *P. aeruginosa*, and *C. albicans* of chitosan/dextran/glycerol containing 1, 5, or 10 wt% Fe_3_O_4_-NPs. High antimicrobial activity was noted for all the tested materials (cell viability reduction > 93%). Nevertheless, the reduction in cell viability of the *C. albicans* strain was slightly lower for the material containing 1 wt% NPs (reached 70%) and exceeded 80% for the 5 and 10 wt% NP-loaded samples. Meanwhile, Arab et al. [83] synthesized PVA hydrogels containing different amounts of ZnO-NPs. Samples containing 0.05, 0.1, and 0.2 wt% of ZnO-NPs were prepared and tested for antibacterial properties. Antibacterial activity estimation was conducted against *B. subtilis.* All applied doses of NPs were effective, and no significant changes in the inhibition zones between samples were noted. Likewise, Majumder et al. [84] produced hydrogel-grafted silk fibroin fabrics with 10 wt% ZnO-NPs. The authors evaluated the bactericidal potential of the produced hydrogel against *E. coli* using the agar disc diffusion method. The silk hydrogel containing ZnO-NPs showed 8 mm of calculated inhibition zone, confirming significantly better antibacterial activity compared to the control material, for which no inhibition zone was noted.

#### 3.2.2. Non-metallic Nanoparticles

While metallic NPs that include both metal and metal oxide NPs have been well-studied and their antibacterial activities have been confirmed by numerous scientific works, the efficacy of non-metallic NPs has been rather understated [89]. The group of non-metallic nanoparticles can be divided into two large subgroups: organic NPs and carbon NPs. Organic NPs include dendrimers, ferritins, micelles, liposomes, and polymer nanoparticles, while carbon NPs can be categorized into fullerenes, graphene, carbon black, carbon nanofibers, carbon nanotubes (CNTs), and sometimes activated carbon [90]. Basically, organic NPs are more sensitive to harsh synthesis conditions, being less stable than inorganic ones, especially at high temperatures, leading to difficulties in production processes. Nevertheless, good antimicrobial activity is observed in the case of quaternary phosphoniums, quaternary ammonium compounds, alkyl pyridiniums, and some polymeric NPs [91,92]. A summary of non-metallic-nanoparticle-loaded wound dressing materials is presented in Table 4.

In Ehterami et al.’s study [93], insulin-delivering chitosan NPs were coated on electrospun poly (ε-caprolactone)/collagen (COLL) nanofibers. Produced potential wound care material was investigated regarding its microbial penetration by placing samples on brain heart infusion (BHI) broth. The number of colonies of microbial contamination in the case of samples with insulin-chitosan NPs revealed the same levels of optical density and bacterial colonies as a negative control. Moreover, materials without NPs were characterized by lower antimicrobial properties, indicating the positive impact of the used NPs. Carbon NPs have become increasingly popular because of their exhibition of bactericidal effects [98]. The antimicrobial activity of carbon-based NPs depends on their size and surface area. Higher antimicrobial activity is observed with smaller nanoparticle sizes and greater surface areas [66]. Faraji et al. modified poly-caprolacton (PCL) nanofiber scaffolds with quercetin-loaded graphene oxide (GO) [94]. According to antibacterial activity evaluation against *S. aureus*, pure PCL material did not show antibacterial activity, whereas bacterial growth on the scaffold with NPs was reduced by 25% compared to PCL and control samples. In addition, further bactericidal effect improvement was noted for material made of PCL/NPs/quercetin (56%). In another study, Jian et al. [95] produced wound dressings made of thermoplastic polyurethane-modified GO (TPU). A comprehensive evaluation of the properties of the produced biomaterials was conducted. Three different concentrations of GO and grafted graphene oxide (MGO) were used: 0.1, 0.5, and 1 wt%. According to the obtained results, the antibacterial properties of the porous membranes increased with the increase in GO and MGO contents. According to the viability test results, the most effective antibacterial concentration was 0.5 wt%. The authors also proved that MGO/thermoplastic polyurethane material possessed better bactericidal activity than GO thermoplastic polyurethane. The same results were noted in fluorescence staining experiments, where after 4 h incubation in GO/TPU, a large number of bacteria were vigorous compared to completely opposite results for MGO/TPU. Xi at al. [96] incorporated aloe-emodin (AE)/carbon NPs into a polyethylene glycol hybrid gel, trying to improve the long-term antibacterial activity of the biomaterial. According to the authors, the biomaterials were evaluated by exposing the hydrogel surfaces to bacterial suspension. All tested hydrogels were separated into two groups, the first of which was additionally treated with 10 min of near-infrared-light (NIR) irradiation to improve the bactericidal effect. Unfortunately, without NIR irradiation, all the materials had no obvious antibacterial activity. The same results were noted for both biomaterials based only on carbon NPs and on a mix of aloe-emodin and carbon NPs, indicating that AE molecules had no antibacterial effect. Nevertheless, in NIR-treated biomaterials, carbon NP-containing hydrogels significantly reduced bacterial viability. Meanwhile, Omidi et al. [97] developed pH-sensitive carbon dots/chitosan hydrogels for wound healing applications. Different concentrations of carbon NPs were used: 0.25, 0.5, 1, and 2 wt%. Antibacterial measurements were conducted against *S. aureus* using disc diffusion and optical density methods. The authors proved the high antibacterial activity and almost complete inhibition of bacterial growth in the case of NPs at doses exceeding 1 wt%. No bactericidal effect of the pure chitosan hydrogel was noted.

### 3.3. Cationic Organic Agent-Loaded Dressing Materials

High amounts of positively charged groups, which are present in cationic organic systems, interact with bacteria that are characterized by negatively charged cell membranes [42]. Thus, the antibacterial effect of cationic organic agents is based on the impact of positively charged groups with the cell’s outer surface membrane’s negatively charged lipid head groups, causing bacterial penetration and further membrane disruption [99]. Among all cationic chemicals, the most frequently used for antimicrobial wound dressing production are chitosan, antimicrobial peptides, and cationic polymers [44]. The cationic nature of chitosan facilitates protonation, endowing its bactericidal effect [100]. Many examples of antimicrobial chitosan-based dressing materials have been described in previous sections; therefore, in order not to repeat information, this section will focus on antimicrobial peptides and other cationic polymers.

#### 3.3.1. Antimicrobial Peptides

Antimicrobial peptides (AMPs) form a large part of innate immunity and are produced by most organisms, including bacteria, protozoa, plants, fungi, vertebrates, and humans [101,102]. These low-molecular-weight molecules (<10 kDa) are composed of up to 100 amino acid residues [102]. Their cationic, hydrophobic, or amphipathic natures predispose diverse biological activities against not only Gram-negative and -positive bacteria, but also fungi, viruses, and even tumors [103]. The latest scientific reports on the evaluation of the antimicrobial properties of dressing materials containing AMPs are presented in Table 5.

Zefeng et al. [104] synthesized antimicrobial peptide-conjugated alginate/hyaluronic acid/collagen (ALG/HA/COL-AMP) porous materials for accelerated infected wound healing. The antimicrobial activity of ALG/HA/COL-AMP dressings was estimated in vitro using an inhibition zone test and a colony-counting test, and in vivo by evaluation of the antimicrobial efficacy of the dressings during the treatment of an infected animal wound. *S. aureus*, MRSA, and *E. coli* were used in the experiments. In the inhibition zone test, biomaterial with AMP revealed 19.4 ± 0.8, 30.3 ± 1.1, and 22.1 ± 0.7 mm inhibition zones against *E. coli*, *S. aureus*, and MRSA, respectively. No bactericidal effect was noted in the case of biomaterial without AMP. In an in vivo evaluation of anti-infective activity, the wound area percentage for the biomaterial with AMP was significantly lower compared to the gauze and ALG/HA/COL samples. Additionally, the bacterial count test for infected wounds revealed significantly lower bacterial numbers for ALG/HA/COL-AMP (45 CFU/wound for *S. aureus* and ~0 CFU/wound for *E. coli*) in comparison to the control gauze group (1.8 × 10^7^ CFU/wound for *S. aureus* and 4.2 × 10^4^ CFU/wound for *E. coli*). Qianwen et al. [105] evaluated the antibacterial properties of HA nanofiber mats loaded with two different concentrations of ε-polylysine (EPL). The authors evaluated the antibacterial activity of the produced materials using the agar diffusion method. Despite the fact that a thin inhibition zone was observed only for material with a lower concentration of EPL, significant bacterial morphological changes were noted during SEM analysis. Both bacterial strains’ morphologies were changed, and the cells were wrinkled with irregular edges. Moreover, completely ruptured cells were noted, indicating the material’s inhibitory effects in direct contact with bacteria. In turn, Amariei et al. [106] investigated the antibacterial activity of PAA/PVA fibrous materials loaded with lysozyme or nisin. The authors observed a strong bactericidal effect of materials containing lysozyme during the agar diffusion test in comparison to the pure material. The inhibition zone increased with incubation time, and no correlation between lysozyme amount and bactericidal effect was noted. In the case of the nisin, a lack of antibacterial activity was observed. Besides the fact that the selected nisin concentrations were comparable to the minimum inhibitory concentration of nisin for *S. aureus* (10 μg/mL), rapid compound diffusion throughout the agar plate could explain the lack of antibacterial activity. In another work [107], the authors estimated the antibacterial activity of polyethylene oxide (PEO) material that contained one of the following two AMPs: GH12-COOH-M2 (type 1 AMP) and AMP2 (type 2 AMP). For this purpose, different concentrations (3.5, 7, 10.5, 14, and 17,5% *w*/*v*) of tested AMPs were added to PEO materials. Antibacterial activity was evaluated in a direct test against *S. epidermidis* using an Alamar Blue assay. Promising bacterial inhibition was noted for biomaterials that contained at least 10.5% *w/v* of the tested type 1 or 2 AMPs. Bacterial viability decreased from approximately 40% (control sample) to 20% in the case of the selected concentration for both AMPs. With increasing preselected compound concentrations, a further decrease in cell viability was observed. In turn, Hizari et al. [108] fabricated silk fibroin/gelatin (SF/Gel) sponge material loaded with various concentrations of a CM_11_ peptide to improve biomaterial antibacterial properties. Three different concentrations were chosen: 0.8, 1.6, and 3.2% *w*/*v*. The antibacterial activity of CM11-loaded materials was tested against standard and resistant strains of *E. coli*, *S. aureus*, and *P. aeruginosa.* In the disc diffusion test, biomaterial without AMP was characterized by a lack of bactericidal effect. Biomaterials with the lowest concentration (0.8% *w*/*v*) of CM_11_ peptide revealed antibacterial activity only in the case of standard strains. Nevertheless, doubling the concentration (1.6% *w*/*v*) caused growth inhibition zones against all tested standard and resistant strains, indicating the great potential of CM_11_ use in infected wound treatment. Razaei et al. [109] modified thermo-responsive chitosan hydrogels (TCTSs) with different concentrations of piscidin-1 (0.4, 0.8, and 1.6% *w*/*v*) to fabricate potential antibacterial wound dressings for use against resistant clinical isolates (*Acinetobacter baumannii*). The antibacterial activity of the synthesized materials was estimated against standard strain and drug-resistant isolates using disc diffusion assays. Pure TCTS did not reveal any antibacterial activity against *A. baumannii*, while all concentrations of the tested AMP loaded into the biomaterial inhibited the growth of standard strain bacteria. Only 1.6% *w/v* of piscidin-1 possessed antibacterial activity against resistant *A. baumannii*.

#### 3.3.2. Cationic Organic Polymers

Natural and synthetic organic polymers that are cationic in character, owing to positively charged groups, exhibit wide-spectrum antibacterial activity [99]. Despite the high bactericidal efficiency of cationic polymers and the lack of bacterial resistance, the complex synthesis and emerging toxicity of produced materials are quite common scientific challenges [44]. The studies that focused on the positive impact of cationic polymers on biomaterials’ antimicrobial properties are summarized in Table 6.

Yang et al. [110] modified polydopamine/polyacrylamide hydrogel (PDA/PAM) with cationic polyelectrolyte brushes grafted from bacterial cellulose nanofibers (BCDs), due to the unsatisfactory antibacterial properties of PDA/PAM. Three different concentrations of BCD (5, 10, and 15 wt%) were added to PDA/PAM, and the antibacterial properties of the biomaterials were tested against *E. coli* and *S. aureus.* The hydrogels were immersed in bacterial culture solution, and optical density (OD) values of the cultured bacteria were measured at different time intervals. For the BCD-free control sample, OD increase was noted after 8 h for *S. aureus* and 6 h for *E. coli.* In contrast to the control, OD values for 10% and 15%BCD-loaded hydrogels did not increase during the experiment. The 5% BCD/PDA/PAM hydrogel revealed OD increase after 24 h for *S. aureus* and 12 h for *E. coli*, indicating low antibacterial activity. Comparable results were noted after live/dead staining of bacteria, where the concentration that started with 10% of BCD showed a highly effective antibacterial effect. In turn, Liang et al. [111] incorporated poly(hexamethylene biguanide) hydrochloride (PHMB) into silk fibroin (SF) sponges to enhance antibacterial function. Antibacterial activity tests of the modified materials were conducted against *E. coli* and *S. aureus.* The authors proved the effectiveness of PHMB at concentrations higher than 2 wt%. Obvious inhibition zones of 10.67 mm and 10.5 mm for *S. aureus* and *E. coli*, respectively, were observed after the increase in cationic polymer concentration to 2 wt%. Further compound concentration increase resulted in better bactericidal effect and inhibition zone widening. In another work [112], the authors used cationic nanofibrillated cellulose (CCNF) to endow a sodium alginate hydrogel (SA) with antibacterial properties. After biomaterial synthesis, a colony-counting method was used to evaluate bactericidal properties. A statistically significant reduction in bacteria colony number was noted for the CCNF-SA sample. The cationic polymer-loaded material possessed an 84.2% antibacterial rate for *B. subtilis* and a rate of 90.3% for *E. coli*. Complete inhibition of bacterial growth was obtained only after the incorporation of tetracycline hydrochloride into the produced biomaterial. Yang et al. [113] synthesized polyvinyl alcohol-formaldehyde (PVF) sponges by grafting [2-(methacryloyloxy) ethyl]-trimethyl ammonium chloride (DMC) onto PVF. Antibacterial properties were estimated for a wide range of DMC concentrations, starting with 5 and ending with 80 wt%. According to the conducted tests, the highest antibacterial activities against *E. coli*, *S. aureus*, and *P. aeruginosa* were noted for 20, 40, and 60 wt% of the cationic polymer, which reduced the initial inoculum concentration of 1.0 × 10^8^ CFU/mL below 1000 CFU/mL. DMC-free PVF material was characterized by almost the same CFU as the initial inoculum concentration.

### 3.4. Natural-Compound-Loaded Biomaterials

Plant-derived compounds, such as monoterpenes, terpenoids, and phenylpropanoids (components of essential oils), polyphenols and plant extracts, and curcumin, have recently been added most often to dressing materials as bioactive agents. Mentioned natural agents possess key features required for the promotion of the regeneration of chronic and infected wounds, i.e., antioxidant, anti-inflammatory, pro-healing, and antimicrobial properties. The following sections summarize the antimicrobial dressing materials produced using naturally derived compounds.

#### 3.4.1. Essential-Oil-Enriched Dressing Materials

Essential oils (EOs) are mixtures of compounds formed as a result of secondary plant metabolism [114]. EOs can be synthesized in various parts of plants: flowers, leaves, roots, seeds, and fruits, and are distinguished by strong smells [114,115]. Essential oils are used in various branches of industry, such as food production, cosmetology, and pharmacy. Due to the many beneficial activities of EOs, such as antibacterial, antiviral, antifungal, insecticidal, analgesic, anticancer, antioxidant, and anti-inflammatory effects, they have attracted great interest in biomedical applications [116]. Antimicrobial properties, as well as the mechanisms of action of EOs, have been clarified in many pioneering works [117]. The latest scientific reports concerning the evaluation of the antimicrobial properties of essential-oil-enriched wound dressing materials are presented in Table 7.

Altaf et al. [118] investigated a hydrogel membrane made of PVA, starch, and glutaraldehyde (cross-linker). The dressings were enriched with oregano oil, clove oil, and tea tree oil. The antibacterial effects of the obtained membranes were examined by the disc diffusion method using Gram-negative *E. coli* and Gram-positive *S. aureus* bacterial strains. The best results were achieved after adding 0.1 mL of clove oil to the biomaterial. Bacterial exposure to the biomaterial with the abovementioned EO resulted in zone growth inhibition of 37 ± 0.29 mm for *E. coli* and 39 ± 0.57 mm for *S. aureus*. On the other hand, oregano oil showed the lowest antibacterial activity for both tested bacterial strains. In another study, Barzegar et al. [119] produced a nanofibrous scaffold with an inner layer composed of chitosan and PVA surrounded by an outer layer of polyvinylpyrrolidone and maltodextrin (MD). The inner layer constituting the core of the dressing contained immobilized essential oil from a plant: *Oliveria decumbens* or *Satureja mutica*. Using the colony-counting method, it was shown that the scaffold itself, probably due to the content of chitosan, had some antibacterial activity against the tested bacterial strains, while it did not reduce the growth of *Candida* strains. However, the presence of *O. decumbens* and *S. mutica* EOs resulted in total inhibition of the growth of *E. coli*, *P. aeruginosa*, *S. aureus*, *Candida dubliniensis*, and *C. albicans* strains. It was assumed that the antimicrobial effects of immobilized EOs may be related to the contents of phenolic compounds, for example, carvacrol. In turn, Hamedi et al. [120] developed a film of chitosan, alginate, and thyme oil nanoemulsion by a casting/solvent evaporation method. Using the viable-cell-counting method, it was observed that the addition of 0.5% thyme oil to the biomaterial caused a significant reduction in the number of *E. coli* and *S. aureus* cells compared to the negative control. Nevertheless, the number of bacterial cells after incubation with the EO-supplemented material was similar to the original number of bacterial cells in the inoculum, meaning that the membrane probably limited the growth of the tested microorganisms. Gheorghita et al. [121], in addition to thyme oil, also incorporated EOs of pine, peppermint, and fennel into PVA- and PVP-based materials. The EOs were loaded into the biomaterials in one of two ways, either by adding the EOs to the mixtures or by adding the microencapsulated EOs. It was noted that the samples containing thyme oil most effectively inhibited the growth of microorganisms: *S. aureus*, *Enterococcus faecalis*, *E. coli*, and *C. albicans*. The exception was the *P. aeruginosa* strain, characterized by high resistance to all tested EOs. Moreover, a higher antimicrobial activity of materials containing microencapsulated thyme oil was observed compared to EOs added to the mixtures. The wound dressing containing pine EO added directly to the mixture showed the lowest inhibitory effect on the tested strains of microorganisms. An extensive screening of many essential oils was also performed by Liakos et al. [115]. EOs of blue chamomile, elicriso italic, cinnamon, tea tree, lavender, peppermint, lemongrass, lemon, and eucalyptus were incorporated into sodium alginate films. Studies showed that wound dressings with tea tree, cinnamon, lemongrass, and peppermint oils achieved the best results. They provided a zone of growth inhibition of *C. albicans* at all three tested concentrations (16, 50, and 66% *w*/*v*), while *E. coli* growth was reduced at the highest and average concentrations used. Only the biomaterial with chamomile blue oil did not have antimicrobial properties against the tested strains at all tested concentrations. Scientists also investigated the benefits of combining the antimicrobial action of essential oils and metal ions. Brindhadevi et al. [114] developed a modification of sodium alginate dressing fabrics with Ag-NPs and labdanum EO (from *Cistus ladanifer*) by an immersion method. Dressings enriched with Ag-NPs and labdanum EO successfully inhibited the growth of the Gram-negative bacteria *Klebsiella pneumoniae* and *E. coli* and the Gram-positive bacteria *B. subtilis* and *S. aureus*, as well as *Aspergillus niger* fungus. The dressings were most effective against the *S. aureus* strain, with the zone of growth inhibition equal to 35 mm for the material with only Ag-NPs and 45 mm for the dressing enriched with both Ag-NPs and EO. Single bioactive compounds derived from essential oils were also used as biomaterial ingredients. Cremar et al. [122] created a biomaterial based on thin chitosan fibers enriched with cinnamaldehyde using a centrifugal spinning method. Cinnamaldehyde is a component of EO derived from cinnamon bark. A study of the antibacterial activity of a dressing containing 0.8% cinnamaldehyde against *S. aureus* showed complete inhibition of pathogen growth with a zone of inhibition of 5 to 10 mm. Interestingly, the bactericidal effect of cinnamaldehyde was comparable to the effect of the biomaterial with Ag-NPs incorporated.

#### 3.4.2. Polyphenol-Enriched Dressing Materials

Polyphenols are compounds commonly obtained from plants and marine organisms. There are two main subsets of polyphenols: flavonoid compounds, such as anthocyanidins; flavonols; and non-flavonoid compounds, such as tannins, lignans, and phenolic acids. Due to the wide range of biological properties, polyphenols are often used in many fields of medicine and pharmacy. In the case of skin regeneration, their antioxidant, pro-healing, and antimicrobial effects are particularly attractive [123]. The latest reports focused on the positive impact of polyphenols on biomaterials’ antibacterial properties are summarized in Table 8.

Li et al. [124] created a porous dressing based on carboxymethyl chitosan and sodium alginate enriched with tea polyphenols (TPs). The antibacterial activity against *E coli* and *S. aureus* of TP-loaded biomaterials was tested before and after impregnation in a solution of CaCl_2_, glycerol, and ethanol. Non-impregnated samples with TP inhibited bacterial growth more effectively than impregnated materials. After 18 h of contact with the material containing TP at a concentration of 1 wt%, decreases in the viability of *E. coli* and *S. aureus* cells by approximately 99.92% and 100%, respectively, were observed. Zeng et al. [125] produced a hydrogel with an immobilized complex made of Cu^2+^ ions and the polyphenol found in the largest amounts in green tea: epigallocatechin-3-gallate (EGCG). The basis of the developed biomaterial for the treatment of chronic wounds was silk fibroin and kappa-carrageenan (kCA). Studies demonstrated that the hydrogel containing only the EGCG compound reduced the growth of bacteria: *S. aureus* and *E. coli* (kill ratio ≈ 43%). However, the antibacterial activity of EGCG increased significantly when it was immersed in a Cu^2+^ solution. A 30 min incubation of the biomaterial in a solution of metal ions resulted in an increase in the killing rate of both tested bacteria to about 93%. The authors suggested that the improved antibacterial properties of the material were due to the sustained release of the polyphenol from the metal complex. Wei et al. [126], apart from EGCG, also tested two other polyphenols: tannic acid (TA) and oligomeric proanthocyanidins (OPCs), and, additionally, the same polyphenols in the form of Cu^2+^ cross-linked nanoparticles. Polyphenols in two forms were introduced into the biomaterial based on carboxymethyl chitosan and phenylboronic acid (PBA). After 18 h exposure of *S. aureus* and *E. coli* to the dressing materials on the agar plates, only a few colonies of bacteria were observed. The antibacterial properties of the wound dressings were confirmed by in vivo tests using the wounds of rats infected with *S. aureus*. The vast majority of bacteria were killed in infected wounds by the use of hydrogels. The authors emphasized that the bactericidal properties of the biomaterials were the result of the synergistic effect of polyphenols, Cu^2+^ ions, and carboxymethyl chitosan. Xu et al. [127] developed a biomaterial in the form of composite nanofibers of chitosan, pullulan, and tannic acid. Tannic acid is a polyphenol commonly found in plants with antioxidant, antibacterial, and strong astringent properties. Using the method of counting live cells, it was shown that the biomaterial containing two compounds with antibacterial activity—chitosan and tannic acid—caused complete inhibition of the growth of Gram-negative *E. coli* bacteria. Cheng et al. [128] also incorporated tannic acid as an antibacterial agent into a PVA and agar-based hydrogel. In vitro tests proved that the biomaterial with the lowest tested concentration of TA (1%) revealed high antibacterial activity against *S. aureus* and *E. coli*. This activity was also confirmed in in vivo studies using a rat wound model infected with the *S. aureus* bacterium. The test results revealed no signs of infection in the wounds of the rats treated with the TA biomaterial, while the wounds of the untreated rats became seriously infected, as indicated by suppuration. Moreover, it was observed that the wounds of rats treated with TA-loaded dressings showed a faster healing process compared to the wounds of untreated rats and those treated with dressings without polyphenol. In another study, Wutticharoenmongkol et al. [129] added gallic acid (GA) to a dressing made of cellulose acetate (CA) nanofibers. GA is a polyphenol commonly found in tea leaves, fruits, vegetables, and nuts. Using the agar disc diffusion method, the bactericidal activity of biomaterials containing 20 wt% and 40 wt% GA against *S. aureus* was tested. Both wound dressings with low and high contents of GA showed antibacterial properties, with the average zones of growth inhibition of *S. aureus* equal to 15.6 mm and 17.5 mm, respectively. Fernandez-Ponce et al. [130] incorporated mango leaf extract into an alginate dressing using supercritical impregnation. It was demonstrated that the main components of the mango leaf extract were polyphenols, such as gallic acid, iriflophenone 3-C-β-D-glucoside, iriflophenone 3-C-(2-O-p-hydroxybenzoyl)-β-D-glucoside, and mangiferin. Next, the antibacterial activity of the biomaterial against *S. aureus* bacteria, which is the most frequently detected pathogen in infected foot wounds of diabetic patients, was tested. The percent of growth inhibition of *S. aureus* after exposure to the impregnated biomaterial was 64.75%. The IC_50_ determined for the mango leaf extract alone was 68.77 ppm. Thus, the bactericidal effect of the extract did not decrease after incorporating it into the dressing material.

#### 3.4.3. Curcumin-Enriched Dressing Materials

Curcumin is a chemical compound obtained from the herb *Curcuma longa*, belonging to the group of curcuminoids that are phenolic pigments. Due to its antimicrobial, antioxidant, anti-inflammatory, and anti-rheumatic properties, curcumin has been the subject of research performed by many scientists. However, the use of curcumin in clinical practice is still a challenge due to its rapid metabolism, poor water solubility, and limited tissue absorption. To facilitate therapeutic use, curcumin is supplied in various forms, for example, in membranes, emulsions, hydrogels, encapsulated in polymer micelles, or in the form of nanoparticles [131,132]. A summary of curcumin-loaded wound dressing materials is presented in Table 9.

Khamrai et al. [133] produced a hydrogel film consisting of gelatin and bacterial cellulose with immobilized curcumin. The research results indicated that the biomaterial containing curcumin inhibited the growth of Gram-positive *S. aureus* and Gram-negative *E. coli* bacteria. The size of the zone of growth inhibition for *S. aureus* and *E. coli* was 19 mm and 15 mm, respectively. In addition, SEM image analysis showed that the *E. coli* bacterium had a shrunken cell wall after contact with the curcumin foil compared to the smooth surface of the cell wall observed in the control. The antibacterial activity of the material with curcumin was also confirmed by fluorescent staining with propidium iodide (PI; stains dead cells) and 4′-6-diamidino-2-phenylindole (DAPI; stains viable and dead cells). It was observed that almost all cells emitted fluorescence characteristics of PI after their exposure to the biomaterial with curcumin. This indicated that the bacterial cells were either dead or had disrupted cell membranes. Similarly, Manna et al. [134] produced a curcumin-loaded biomaterial in the form of films based on carboxymethyl guar gum and gelatin. The wound dressing with curcumin effectively inhibited, to similar extents, the growth of the Gram-positive bacteria *Bacillus cereus*, *Lysinibacillus*, *B. subtilis*, and *S. aureus* (inhibition zone ranges: 12–16 mm) and the Gram-negative bacteria *Enterobacter aerogenes*, *E. coli*, *P. aeruginosa*, and *Vibrio vulnificus* (inhibition zone ranges: 15–17 mm). Tong et al. [135] developed a nanocrystalline cellulose film for curcumin delivery dressings in the treatment of diabetic wounds. The material with immobilized curcumin inhibited the growth of the bacteria *Bacillus coagulans*, MRSA, *Streptococcus* sp., *E. coli*, and *Proteus mirabilis* and the yeast *C. albicans*. The highest antimicrobial activity was observed against *B. coagulans*, with the zone of growth inhibition equal to 67 mm. Nevertheless, the results of the Hohenstein challenge test confirmed a decrease in the growth of all tested strains of microorganisms, in most cases even up to 99%, compared to the control. Due to the controlled release of the active substance by the dressing, no re-growth of microorganisms (bacteria or yeast) was noted during the experiment. In another study, Tsekova et al. [136] optimized the curcumin release profile from the wound dressing through an assortment of composition and material manufacturing techniques. Different configurations of the polymer matrix made of cellulose acetate (CA) and PVP were produced by two methods: dual spinneret electrospinning and one-pot electrospinning. In addition, the material was irradiated with blue light during microbiological tests, since, as a result of curcumin irradiation, reactive oxygen species, toxic to bacteria, were created. Research showed that 2 h exposure to biomaterials containing curcumin resulted in a significant reduction in the number of viable cells of the *S. aureus* strain. The dressing material consisting of CA and PVP enriched with curcumin produced by the dual-spinneret electrospinning method (Curc/CA + Curc/PVP) was characterized by the highest antibacterial activity. A 4 h exposure to this biomaterial resulted in the death of all bacterial cells, possibly due to the most efficient release of curcumin (68%) compared to other tested materials. Moreover, the authors assessed the adhesion of bacterial cells to the surfaces of the materials. Non-adhesive *S. aureus* cells were observed on the surface of the Curc/CA + Curc/PVP material, in contrast to the CA material without curcumin, which allowed bacterial adhesion. In turn, Venkatasubbu and Anusuya [137] covered a cotton fabric with a composite based on PVA, silver, and curcumin nanoparticles. Then, the obtained biomaterials with curcumin at concentrations of 250, 500, 700, and 1000 μg/mL were tested against a wide spectrum of bacterial strains using the agar diffusion method. The study showed that curcumin inhibited the growth of all tested bacteria: *E. coli*, *S. aureus*, *B. subtilis*, *Proteus vulgaris*, *S. epidermis*, *E. faecalis*, *K. pneumoniae*, *Pseudomonas mendocina*, *E. aerogenes*, and *Coliform*. The antibacterial activity of curcumin increased proportionally to its concentration in the biomaterials. In addition, the effect of curcumin was enhanced by silver ions released from the nanocomposite. The authors emphasized that the reduction in the size of curcumin particles to the nanoform resulted in improved solubility of the compound in water and additionally increased its penetration and uptake by bacterial cells. Sadeghianmaryan et al. [132] also observed a synergistic antibacterial effect of curcumin and quaternary ammonium salt-modified montmorillonite (MMT). Using the electrospinning method, they produced a curcumin-loaded nanocomposite material consisting of PCL and MMT. The test results revealed a reduction in the number of *E. coli* and *S. aureus* colonies after exposure to the PCL/MMT biomaterial in the case of lower concentrations of bacteria. The addition of curcumin resulted in the highest decrease in the number of bacterial colonies (by over 50%). The antibacterial activity of the biomaterial was additionally confirmed in an MTT test. When exposed to the PCL/MMT/curcumin biomaterial, the cell viability of *S. aureus* and *E. coli* decreased by an average of 54% and 46%, respectively. Ramalingam et al. [138] studied the antibacterial activity of curcumin against multidrug-resistant bacteria. The compound was immobilized in poly(2-hydroxyethyl methacrylate) p(HEMA) nanofiber material produced by electrospinning. The formation of growth inhibition zones of approximately 17 mm for MRSA and non-MRSA bacteria was observed after contact with biomaterials containing curcumin. The wound dressing was also effective against extended-spectrum β lactamse (ESBL)-producing *E. coli* and *K. pneumonia* and non-ESBL-producing *E. coli* and *K. pneumonia* (zones of growth inhibition were approx. 18 mm). Niranjan et al. [139] assessed the antibacterial effect of patches consisting of PVA, chitosan, and nanocurcumin against strains of microorganisms usually inhabiting wounds. The zones of growth inhibition for *S. aureus*, *B. subtilis*, *E. coli*, and *P. aeruginosa* that resulted from the presence of the patches were 15 mm, 14 mm, 18 mm, and 20 mm, respectively. The authors emphasized that curcumin’s effectiveness was associated with its nanosize, which increased its bioavailability.

## 4. Clinical Use of Antimicrobial Dressings

The process from the discovery of a therapeutic agent to clinical trials and then market launch is incredibly long and expensive. Achieving this goal requires not only a huge financial, scientific, and technological background, but also perseverance and luck. Only a small percentage of therapeutic agents are approved and released for trade [140]. Gottrup et al. [141] compared treatment with collagen, oxidized regenerated cellulose (ORC), and silver dressings with traditional treatments in patients with diabetic foot ulcers. The study group included 39 patients. The silver-containing dressing was applied directly to the wound bed. It was observed that the dressing made of collagen, ORC, and silver accelerated regeneration and prevented wound infection compared to the control group. Among the participants treated with the silver dressing, no patient was withdrawn due to infection, while in the control group, 31% of patients were affected by this problem. In turn, Wu et al. [142] studied the effect of a nanosilver dressing compared to sulfadiazine silver cream in the treatment of infected second-degree burn wounds. After 14 days of medication, a bacteriological culture of exudate from the patients’ wounds was carried out and the grown bacterial species were identified. The use of a dressing with nanosilver resulted in a reduction in positive bacterial cultures. In addition, a shortened period of wound healing and loss of pigmentation was noted, as well as a reduced level of the pro-inflammatory cytokine IL-1β. In another article, Wang. et al. [143] investigated the antimicrobial activity of a chitosan dressing. The study involved patients with postoperative wounds of the abdominal cavity; the control group consisted of patients with gauze dressings. The results of a clinical study revealed that the application of a dressing with chitosan resulted in inhibition of the growth of pathogenic bacteria up to 8 days after surgery. The dressing with chitosan showed antibacterial activity against, bacteria from the *Enterobacteriaceae*, *Aeromonadaceae*, and *Muribaculaceae* families, among others. In addition, after applying the dressing with chitosan, growth of probiotic bacteria—for example, *Prevotella*, *Oscillibacter* and *Lactobacillus*—was observed. Uckay et al. [144] evaluated the effect of collagen sponge with gentamicin on patients with infected diabetic foot ulcers in combination with systemically administered antibiotics. The control group consisted of patients treated only with antibiotics. The results of the study showed that the use of a dressing with gentamicin did not significantly improve wound healing compared to the control group. However, it was noted that the gentamicin sponges were well tolerated by the patient’s body. Similar results were obtained in other research, where a collagen sponge with gentamicin was used in the treated group, but without additional antibiotics [145]. In this case, there were also no differences between the patients treated with the gentamicin dressing and the control group. Sibbald et al. [146] treated patients with foot or leg ulcers with a polyhexamethylene biguanide (PHMB) foam dressing. To patients from the control group a foam dressing was applied without an antibacterial agent. The results demonstrated that the use of the PHMB dressing significantly reduced wound infection compared to the control dressing. After 4 weeks, the appearance of microorganisms was observed in 5.3% of wounds treated with the PHMB dressing and in 33% of wounds in the control group.

## 5. Conclusions

The present review article has described the latest reports on the improvement of the antimicrobial properties of potential wound dressing materials using antibiotics, nanoparticles, and naturally derived active compounds. The variety of potential bactericidal compounds described in the present work provides precious scientific knowledge that may be used for the modification of wound dressing materials to achieve antimicrobial activity. The review article provides adequate knowledge on the antimicrobial effectiveness of appropriate concentrations of bioactive compounds and allows the faster planning of research based on the information contained herein. Based on the information found in the literature, it may be seen that researchers have applied a wide range of concentrations of the same bioactive compounds, obtaining different results. However, it is worth noting that a large range of applied compound concentrations may result not only from the chemical compositions of the dressings, but also from the described methods used in their production. An extensive review of the literature confirmed that, despite the frequent use and effectiveness of antibiotics, scientists are still searching for an alternative to the use of antibiotic therapy to avoid the problem of an increasing number of drug-resistant strains. Nevertheless, despite many scientific contributions related to the development of antibiotic-free antimicrobial wound dressings, these novel materials are often not introduced to the market, so the problem of heavily infected chronic wounds still remains a major challenge for physicians. In view of the above, it seems reasonable to continue research on modifications of dressing materials in order to accelerate the healing of infected wounds, as well as to move the research to the next stage, namely, the assessment of antimicrobial dressing materials in clinical trials, so that the translation of the scientific studies to clinical practice will be possible.

## Figures and Tables

**Table 1:** Comparison of the effects of bacterial contamination and bacterial infection on wound healing.

Phase of Wound Healing	Effect of Bacterial Contamination	Effect of Bacterial Infection	Ref.
Homeostasis	Unaffected initial wound closure	Impaired healing due to the release of endotoxins, exotoxins, and tissue-destroying enzymes	[16]
Inflammation	Enhanced inflammatory cell accumulation and bactericidal and chemotactic activity	Reduced effectiveness of the complement cascade by depletion of different factors; Increased production of proteolytic enzymes, antimicrobial proteins, and reactive oxygen species; Damage to the host tissue due to increased production of microbicidal molecules synthesized by neutrophils; Prolonged inflammatory state that results in high levels of pro-inflammatory mediators and impaired repair; Disbalance in MMPs and their inhibitor concentrations, contributing to wound chronicity	[17,18,19,20,22]
Proliferation	Unaffected epithelialization and granulation processes	Suppression of endothelial cell migration and proliferation; Biofilm formation due to considerable bacterial colonization over 10^5^ and consequently decreased epithelialization	[11,25,29]
Remodeling	Unaffected tensile strength of the skin as a result of normal healing	Hindered fibroblast replication, and thus limited type I collagen production; Increased production of collagen-digesting enzymes due to the presence of endotoxins	[20,34]

**Table 2:** Antibiotic-loaded biomaterials for wound healing applications.

Antibiotic	Concentration	Type of Wound Dressing	Biomaterial Composition	Tested Microorganism	Ref.
Gentamicin	0.2% wt%	Sponge	Curdlan/agarose	*Staphylococcus aureus*; *Pseudomonas aeruginosa*	[48]
Gentamicin	0–10 wt%	Nanofibers	Chitosan/alginate	*Staphylococcus aureus*; *Escherichia coli*	[60]
Gentamicin	15 wt%	Film	Natural rubber/triethyl citrate/xanthan gum	*Staphylococcus aureus*; *Pseudomonas aeruginosa*	[61]
Tetracycline hydrochloride	0.5 wt%	Sponge	Chitosan, chitosan/aloe vera	*Bacillus subtilis*; *Staphylococus aureus*; *Escherchia coli*; *Klebsiella pnemoniae*	[62]
Tetracycline hydrochloride	0.5% *w*/*v*	Nanofibers	Polyvinyl alcohol/chitosan	*Staphylococcus aureus*; *Staphylococcus epidermidis*; *Escherichia coli*	[63]
Ciprofloxacin	10 wt%	Nanofibers	Poly(N-isopropyl-acrylamide-co-acrylamide)/ polycaprolactone	*Staphylococcus epidermidis*; *Escherichia coli*	[54]
Ciprofloxacin	0.1 wt%	Film	Chitosan/cellulose	*Pseudomonas aeruginosa*; *Staphylococcus aureus*	[55]
Silver sulfadiazine	0.125–0.5 wt%	Nanofibers	Cellulose acetate/silver-sulfadiazine	*Escherichia coli*; *Bacillus subtilis*	[56]
Silver sulfadiazine/zeolite complex	5 wt%	Film	Chitosan/zeolite	*Escherichia coli*; *Staphylococcus aureus*; *Pseudomonas aeruginosa*; *Candida albicans*	[57]
Silver sulfadiazine	0.3–0.6 wt%	Nanofibers	Zein	*Escherichia coli*; *Bacillus subtilis*	[59]
Vancomycin	Not provided	Hydrogel	Chitosan/polyvinyl alcohol/polyethylene glycol	*Staphylococcus aureus*	[64]
Ampicillin	2–6 wt%	Hydrogel	Gelatin/gellan gum	*Escherichia coli*; *Staphylococcus aureus*	[65]

**Table 3:** Antimicrobial metallic-nanoparticle-loaded biomaterials for wound healing applications.

Metallic Nanoparticles	Concentration	Type of Wound Dressing	Biomaterial Composition	Tested Microorganism	Ref.
Ag	0.2–0.7 wt%	Nanofibers	Polyvinyl alcohol/ polyvinylpyrrolidone/pectin/mafenide acetate	*Escherichia coli*; *Staphylococcus aureus*; *Pseudomonas aeruginosa*	[74]
Ag	Not provided	Sponge	Chitosan	*Escherichia coli*; *Staphylococcus aureus*; *Pseudomonas aeruginosa*	[75]
Ag	<0.1 wt%	Foam	Lignin-based/polyurethane	*Escherichia coli*; *Staphylococcus aureus*	[76]
Au	Not provided	Hydrogel	Polyacrylic acid/polyallylamine hydrochloride/poly ethylene glycol	*Staphylococcus aureus*; *Pseudomonas aeruginosa*	[77]
Au	≈4 × 10^−4^ wt%	Gel	Pluronic^®^F127/hydroxypropyl methylcellulose	*Staphylococcus aureus*	[78]
CuO	0.05–0.1 wt%	Film	Polycaprolactone	Methicillin-resistant *Staphylococcus aureus*	[79]
CuO	1 wt%	Nanofibers	Polycaprolactone/gelatin	*Escherichia coli*; *Pseudomonas aeruginosa*; Methicillin-resistant *Staphylococcus aureus*; *Staphylococcus aureus*	[80]
Fe_3_O_4_	5–15 wt%	Hydrogel	Poly(hydroxyl ethyl methacrylate)	*Escherichia coli*; *Staphylococcus aureus*	[81]
Fe_3_O_4_	1–10 wt%	Hydrogel	Chitosan/dextran/glycerol	*Staphylococcus aureus*; *Pseudomonas aeruginosa*; *Candida albicans*	[82]
ZnO	0.05–0.2 wt%	Hydrogel	Polyvinyl alcohol	*Bacillus subtilis*	[83]
ZnO	10 wt%	Hydrogel	Silk woven fabric/ammonium persulphate/N,N′-bismethylacrylamide	*Escherichia coli*	[84]

**Table 4:** Antibacterial non-metallic-nanoparticle-loaded biomaterials for wound healing applications.

Organic Nanoparticles	Concentration	Type of Wound Dressing	Biomaterial Composition	Tested Microorganism	Ref.
Insulin-loaded chitosan	Not provided	Nanofibers	Poly(ε-caprolactone)/collagen	Not provided	[93]
Quercetin-loaded graphene oxide	Not provided	Nanofibers	Poly(ε-caprolactone)/quercetin	*Staphylococcus aureus*	[94]
Graphene oxide/grafted graphene oxide	0.1–1 wt%	Film	Thermoplastic polyurethane	*Staphylococcus aureus*; *Escherichia coli*	[95]
Aloe-Emodin/Carbon	Not provided	Hydrogel	Polyethylene glycol	*Staphylococcus aureus*; *Escherichia coli*	[96]
Carbon dots	0.25–2 wt%	Hydrogel	Chitosan	*Staphylococcus aureus*	[97]

**Table 5:** Antibacterial AMP-loaded biomaterials for wound healing applications.

Antibacterial Peptides	Concentration	Type of Wound Dressing	Biomaterial Composition	Tested Microorganism	Ref.
Tet213	0.05% *w*/*v*	Foam	Alginate/hyaluronic acid/collagen	*Escherichia coli*; *Staphylococcus aureus*	[104]
ε-polylysine	24.6% wt% 27.9% wt%	Nanofiber	Hyaluronic acid	*Escherichia coli*; *Staphylococcus aureus*	[105]
Lysozyme/nisin	1.14 × 10^−5^–8.97 × 10^−6^ mmol/mg	Fibrous material	Polyvinyl alcohol/polyacrylic acid	*Staphylococcus aureus*	[106]
GH12-COOH-M2/ AMP2	3.5–17.5% *w*/*v*	Nanofiber	Polyethylene oxide	*Staphylococcus* *epidermidis*	[107]
CM_11_	0.8, 1.6, and 3.2% *w*/*v*	Sponge	Silk fibroin/gelatin	*Staphylococcus aureus*; *Escherichia coli*; *Pseudomonas aeruginosa*	[108]
Piscidin-1	0.4, 0.8, and 1.6% *w*/*v*	Hydrogel	Chitosan	*Acinetobacter* *baumannii*	[109]

**Table 6:** Antibacterial cationic-polymer-loaded biomaterials for wound healing applications.

Cationic Polymers	Concentration	Type of Wound Dressing	Biomaterial Composition	Tested Microorganism	Ref.
Polydiallyl dimethyl ammonium chloride brushes grafted from bacterial cellulose nanofibers	5, 10, and 15 wt%	Hydrogel	Polydopamine/polyacrylamide	*Escherichia coli*; *Staphylococcus aureus*	[110]
Poly(hexamethylene biguanide) hydrochloride	0.5–10 wt%	Sponge	Silk fibroin	*Escherichia coli*; *Staphylococcus aureus*	[111]
Cationic nanofibrillated cellulose	1.4 wt%	Hydrogel	Sodium alginate	*Bacillus subtilis*; *Escherichia coli*	[112]
[2-(methacryloyloxy)ethyl]trimethyl ammonium chloride	5–80 wt%	Hydrogel	Polyvinyl alcohol-formaldehyde	*Escherichia coli*; *Staphylococcus aureus*; *Pseudomonas* *aeruginosa*	[113]

**Table 7:** Antimicrobial essential-oil-loaded biomaterials for wound healing applications.

Essential Oils or Their Compounds	Concentration	Type of Wound Dressing	Biomaterial Composition	Tested Microorganism	Ref.
Clove, oregano, and tea tree essential oils	Not provided	Hydrogel membrane	Polyvinyl alcohol/starch/glutaraldehyde (cross-linker)	*Escherichia coli*; *Staphylococcus* *aureus*	[118]
*Satureja mutica*, *Oliveria decumbens* essential oils	10 wt%	Nanofibers	Chitosan/polyvinyl alcohol (the core) and poly-vinylpyrrolidone/maltodextrin (the shell)	*Pseudomonas aeruginosa*; *Escherichia coli*; *Staphylococcus aureus*; *Candida dubliniensis*; *Candida albicans*	[119]
Thyme essential oil	1–3% *v*/*v*	Film	Chitosan/alginate	*Escherichia coli*; *Staphylococcus* *aureus*	[120]
Fennel, pine, peppermint, and thyme essential oils	12%	Film	Polyvinyl alcohol/polyvinyl pyrrolidone	*Staphylococcus* *aureus*; *Enterococcus faecalis*; *Escherichia coli*; *Pseudomonas * *aeruginosa*; *Candida albicans*	[121]
Chamomile blue, cinnamon, lavender, tea tree, peppermint, eucalyptus, lemongrass, and lemon essential oils	16, 50, and 66 wt%	Film	Sodium alginate	*Escherichia coli*; *Candida albicans*	[115]
*Cistus ladanifer* essential oils	Not provided	Wound fabric modified by dip coating method	Sodium alginate/silver nanoparticles	*Escherichia coli*; *Klebsiella pneumoniae*; *Staphylococcus aureus*; *Bacillus subtilis*; *Aspergillus niger*	[114]
Cinnamaldehyde	0.8 wt%	Nanofibers	Chitosan	*Staphylococcus aureus*	[122]

**Table 8:** Antibacterial polyphenol-loaded biomaterials for wound healing applications.

Polyphenols	Concentration	Type of Wound Dressing	Biomaterial Composition	Tested Microorganism	Ref.
Tea polyphenols	0.5 and 1 wt%	Foam	Carboxymethyl chitosan/sodium alginate	*Staphylococcus* *aureus*; *Escherichia coli*	[124]
Epigallocatechin-3-gallate	2 wt%	Hydrogel	Silk fibroin/kappa-carrageenan	*Escherichia coli*; *Staphylococcus* *aureus*	[125]
Tannic acid, oligomeric proanthocyanidins (−)-epigallocatechin-3-O-gallate	Not provided	Hydrogel	Carboxymethyl chitosan/phenylboronic acid	*Staphylococcus* *aureus*; *Escherichia coli*	[126]
Tannic acid	1 wt%	Nanofibers	Chitosan/pullulan	*Escherichia coli*	[127]
Tannic acid	1–10% *w*/*v*	Hydrogel	Agar/polyvinyl alcohol	*Staphylococcus* *aureus*; *Escherichia coli*	[128]
Gallic acid	20 and 40 wt%	Nanofibers	Cellulose acetate	*Staphylococcus* *aureus*	[129]
Mango leaf extract (main components: gallic acid, iriflophenone 3-C-β-D-glucoside, iriflophenone 3-C-(2-O-p-hydroxybenzoyl)-β-D-glucoside, and mangiferin)	5% *v*/*v*	Fibrous material	Alginate	*Staphylococcus* *aureus*	[130]

**Table 9:** Antimicrobial curcumin-loaded biomaterials for wound healing applications.

Curcumin Concentration	Type of Wound Dressing	Biomaterial Composition	Tested Microorganism	Ref.
7 and 13 wt%	Hydrogel film	Gelatin/ionically modified bacterial cellulose	*Staphylococcus aureus*; *Escherichia coli*	[133]
1% *w/v*	Film	Carboxylmethyl guar gum/gelatin	*Escherichia coli*; *Enterobacter aerogenes*; *Vibrio vulnificus*; *Pseudomonas aeruginosa*; *Bacillus cereus*; *Bacillus subtilis*; *Lysinibacillus*; *Staphylococcus aureus*	[134]
Not provided	Film	Nanocellulose fibers/polyvinyl alcohol	*Bacillus cereus*; *Bacillus coagulans*; *Streptococcus* sp.; methicilin-resistant *Staphylococcus aureus*; *Escherichia coli*; *Proteus mirabilis*; *Yersinia* sp.; *Pseudomonas aeruginosa*; *Candida albicans*; *Candida utilis*	[135]
10 wt%	Fibrous materials	Cellulose acetate/polyvinylpyrrolidone	*Staphylococcus aureus*	[136]
0.025–0.1 wt%	Cotton cloth	Cotton cloth/polyvinyl alcohol	*Escherichia coli*; *Bacillus subtilis*; *Staphylococcus aureus*; *Proteus* *vulgaris*; *Enterococcusi faecalis*; *Staphylococcus epidermis*; *Klebsiella pneumoniae*; *Enterobacter aerogenes*; *Pseudomonas mendocina*; *Coliform*	[137]
Not provided	Fibrous material	Polycaprolactone/quaternary ammonium salt-modified montmorillonite	*Escherichia coli*; *Staphylococcus aureus*	[132]
3 and 5 wt%	Nanofiber material	Poly(2-hydroxyethyl methacrylate	Methicillin-resistant *staphylococcus aureus*; non-methicillin-resistant *Staphylococcus aureus*; extended-spectrum β-lactamse *Escherichia coli* and *Klebsiella pneumonia*; non-extended-spectrum β-lactamse *Escherichia coli* and *Klebsiella pneumonia*	[138]
Not provided	Transdermal patch	Polyvinyl alcohol/chitosan	*Escherichia coli*, *Pseudomonas* *aeruginosa*; *Staphylococcus aureus*; *Bacillus subtilis*	[139]

## Data Availability

Not applicable.
